# Metabolomics of the effects of Yishenjiangya granules in older adults with hypertension

**DOI:** 10.3389/fphar.2025.1491935

**Published:** 2025-03-11

**Authors:** Yongbo Ma, Yingying Liu, Li Zhuang, Xia Dai, Li Yao, Jie Yu, Lei Zhang

**Affiliations:** ^1^ Shandong University of Traditional Chinese Medicine, Jinan, Shandong, China; ^2^ Shanghai University of Traditional Chinese Medicine, Shanghai, China; ^3^ Affiliated Hospital of Shandong University of Traditional Chinese Medicine, Jinan, Shandong, China; ^4^ Shanghai Changning Tianshan Traditional Chinese Medicine Hospital, Jinan, Shandong, China

**Keywords:** hypertension, Yishenjiangya granules, metabolomics, linoleic acid, arachidonic acid, glycerophospholipid

## Abstract

**Introduction:**

Older adults are a high-risk group for hypertension, with specific characteristics regarding symptoms and treatment. Yishenjiangya granules (YJG), a traditional Chinese medicinal decoction, are widely used to reduce blood pressure and improve clinical symptoms. This study aimed to use metabolomics to explore the clinical effects and underlying mechanisms of YJG in hypertension in older adults.

**Methods:**

The study enrolled patients aged ≥65 years, with systolic blood pressure ≥140 mmHg and/or diastolic blood pressure ≥90 mmHg in sitting positions on different days; the control group comprised 30 healthy participants with normal blood pressure and biochemistry indicators. Ultra-performance liquid chromatography coupled with quadrupole time-of-flight mass spectrometry (UPLC-Q-TOF-MS) was used to analyze plasma metabolites in patients with hypertension before and after YJG intervention.

**Results:**

After YJG treatment, blood pressure decreased significantly; some metabolites showed a trend approaching the control group. UPLC-Q-TOF-MS analysis identified 30 YJG-targeted plasma metabolites in older adult patients with hypertension, including three major metabolic pathways: linoleic acid, arachidonic acid, and glycerophospholipid metabolism.

**Conclusion:**

This study identified that metabolite changes may underlie the clinical mechanism of YJG in treating older adult patients with hypertension, providing a basis for further treatment of hypertension.

## Introduction

Hypertension is the leading cause of cardiovascular disease and premature death worldwide (Mills et al., 2020; Zhou et al., 2021). The number of people aged 30–79 years with hypertension doubled from 1990 to 2019 (Ncd-Risc, 2021). Older adults are more likely to suffer from hypertension because of the interaction between lifestyle and genetic factors (Wang et al., 2023). Hypertension management strategies in older adults must be individualized to consider the degree of frailty, increasingly complex medical comorbidities, and psychosocial factors (Oliveros et al., 2019).

The main treatment for hypertension is oral medications, including angiotensin-converting enzyme inhibitors, angiotensin II receptor antagonists, β receptor blockers, calcium channel blockers, and diuretics, which significantly reduce the risk of cardiovascular diseases and death in different populations. However, these drugs lack specificity for the characteristics of older adults, only weakly improve clinical symptoms, and have many adverse reactions. Traditional Chinese medicine (TCM) has become a complementary and alternative method for the primary and secondary prevention of cardiovascular diseases (Zhao et al., 2018). The potential benefits include reducing blood pressure, improving overall efficacy, improving clinical symptoms, and reducing adverse events (Zhang et al., 2020). A meta-analysis of 2024 patients found that tonifying the kidney TCM showed good outcomes in lowering blood pressure, lowering blood lipids, and improving vascular endothelial functions and clinical symptoms (Wang et al., 2022). Yishenjiangya granules (YJG), TCM for tonifying the kidney, have been used to treat older adults with hypertension in the clinic, achieving good therapeutic effects (Hu et al., 2010). However, because of the complex chemical components of TCM, its mechanism of action remains unclear.

Metabolomics uses high-resolution, highly sensitive, and high-throughput instrumental analysis to qualitatively or quantitatively study metabolites and elucidate metabolic pathways in the body. In recent years, metabolomics studies have provided greater insights into identifying disease-specific biomarkers, predicting treatment outcomes, and monitoring drug safety and efficacy. Patients with hypertension have multiple differential metabolites that affect amino acid metabolism, fatty acid metabolism, and steroid hormone biosynthesis (Zhao et al., 2018). These metabolic changes may impair insulin resistance, inflammatory status, and NO production, ultimately leading to the development and progression of essential hypertension (Deng et al., 2021). In addition, the metabolites and metabolic pathways affected by various drugs may differ, providing a basis for selecting antihypertensive drugs for patients. The composition of TCM is complex; metabolomics research on treating hypertension with TCM is gradually emerging to help explore its mechanism of action (Dong et al., 2020).

This study aimed to investigate the effects of YJG in older adults with hypertension and determine the plasma metabolic profile variations from oral administration. We used ultra-performance liquid chromatography coupled with quadrupole time-of-flight mass spectrometry (UPLC-Q-TOF-MS)-based metabolomics analysis to clarify the underlying hypotensive mechanism, improve the early diagnosis of essential hypertension, and identify novel therapeutic targets.

## Materials and methods

### Chemicals and reagents

Chromatographic grade methanol, formic acid, and acetonitrile were obtained from the Fisher Company, USA. The internal standard was 2-chloro-L-phenylalanine obtained from Macklin Biochemical Co., Ltd. (Shanghai, China). Levoamlodipine maleate tablets (LMTs) were provided by Shijiazhuang Ouyi Pharmaceutical Co., Ltd (Shijiazhuang, China). YJGs were provided by the Pharmacy Department of the Affiliated Hospital of Shandong University of TCM (Jinan, China), comprising *Viscum coloratum* (Kom.), Nakai [Santalaceae], *Ligustrum lucidum* W.T. Aiton [Oleaceae], *Epimedium sagittatum* (Siebold & Zucc.) Maxim. [Berberidaceae], *Astragalus mongholicus* Bunge [Fabaceae], *A. plantagoaquatica* subsp. *orientale* (Sam.) Sam. [Alismataceae], Ziziphus jujuba Mill. [Rhamnaceae], *Achyranthes bidentata* Blume [Amaranthaceae], and *Polygonatum kingianum* Coll.et Hemsl.

### Study participants

This study was conducted between June 2017 and June 2018 at the Affiliated Hospital of Shandong University of TCM. The enrolled patients were aged 65–80 years and diagnosed with Grade 1 or 2 essential hypertension. The diagnostic criteria for hypertension in older adults refer to the “Guidelines for the Prevention and Treatment of Hypertension” (Whelton et al., 2018). The criteria for adults aged ≥65 years stipulate that systolic blood pressure (SBP) ≥140 mmHg and/or diastolic blood pressure (DBP) ≥90 mmHg in sitting positions on different days. Exclusion criteria included a history of drug allergies, moderate or severe diabetes, previous cardiovascular events (myocardial infarction, unstable angina pectoris, or cerebrovascular accident in the last 6 months), and other severe diseases, including combined kidney and liver damage. The control group comprised 30 healthy participants with normal blood pressure, normal blood biochemical indicators, and no other diseases affecting the test results. Baseline data on the participants, including sex, height, and weight, was recorded uniformly. This study was approved by the Ethics Committee of the Affiliated Hospital of the Shandong University of TCM and conducted under supervision (Ethics batch No. 2017020-KY). All participants provided written informed consent.

### Study design

Older adults with hypertension were divided into two groups of 30 cases each. Antihypertensive therapy was discontinued, and strict lifestyle intervention was adopted for 2 weeks before the trial; clinical trials were conducted when blood pressure rose to or exceeded the previous maximum blood pressure level. YJGs were used for intervention in group 1 for 12 weeks; 6 g/bag were taken with warm water, 1 bag/time, 2/day. LMT was used for intervention in group 2 for 12 weeks (2.5 mg/tablet, oral, 1–2 tablets/time, 1/day). Other antihypertensive drugs and measures were discontinued during treatment to eliminate interference from drug factors.

### Blood pressure measurement

This study used a dynamic blood pressure monitor (SpaceLabs 90217, Redmond, USA) for fully automated blood pressure monitoring. The cuff is fixed to the patient’s upper arm by professionals, with the lower edge 2cm∼3 cm away from the elbow socket. The sensing indicator points to the pulsation of the brachial artery, and the tightness is limited to accommodating two fingers. Adjust monitoring interval: During the day (08:00∼22:00), measure once every 30 min; At night (22:00∼08:00 the next day), measure once every hour. The effective monitoring frequency within 24 h shall not be less than 80% of the total blood pressure acquisition frequency. During the monitoring period, there shall be no restrictions on the daily activities of the subjects. To ensure the success rate, the tested patients are required to adopt a sitting position as much as possible during blood pressure measurement, and avoid emotional excitement and vigorous activities. The tested upper limbs shall be kept relatively fixed (Whelton et al., 2018).

### Sample preparation

The participants’ fasting venous blood was collected in the morning before and after the experiment; 5 mL was collected using a disposable negative-pressure anticoagulant collection vessel, which was stood for 1 h and centrifuged at 3500 rpm for 10 min at 4°C. The supernatant was centrifuged at 10,000 rpm for 5 min at 4°C. The plasma was divided into 1.5 mL Eppendorf tubes and stored at −80°C for later use.

The plasma was thawed at 4°C and shaken well for sample preparation. We added 400 μL of acetonitrile and 20 μL internal standard solution (0.05% chloro-L phenylalanine methanol solution) to 100 μL of plasma and vortexed for 2 min. After being placed at 4°C for 6 h, the samples were centrifuged at 12,000 rpm for 15 min at 4°C. After drying with nitrogen, the solid pellet was dissolved in 100 μL of the initial flow phase (2% (v/v) acetonitrile/H_2_O and vortexed for 1 min. After being centrifuged at 12,000 rpm for 15 min, 80 μL of the supernatant filtered through a 0.22 µm cellulose acetate membrane was used for LC–MS analysis. In addition, 20 μL of plasma from each sample was used to prepare quality control (QC) samples to validate the experimental precision, repeatability, and stability.

### UPLC-Q-TOF-MS profiling

Metabolites were detected and identified using a UPLC-Q-TOF-MS system, comprising an UltiMate 3000 ultra-high performance liquid chromatography (UPLC, Thermo Scientific) and a four-stage rod-electrostatic field orbital trap ultra-high resolution mass spectrometer (Q ActiveHybrid Quadrupole-Orbitrap mass spectrometer). A Thermo-C18 column (HypersilGOLDAQ, 100 × 2.1 mm, 1.9 μm) was used for chromatographic separation. The flow rate was 0.30 mL/min. The column temperature was 45°C; the injector temperature was 15°C; 6 μL samples were tested per injection. The gradient elution method was adopted, and the initial mobile phase was equilibrated for 5 min before each injection. Mobile phase A was 0.05% (v/v) formic acid in water; mobile phase B was 0.05% (v/v) formic acid in acetonitrile. The gradient elution was 2% B at 0–1 min, 2%–40% B at 1–6 min, and 40%–98% B at 6–17 min.

The positive- and negative-ion modes were detected using mass spectrometry detection conditions: ion source high energy spark-induced breakdown lonization, capillary voltage 3,500 V, capillary temperature 320°C, source temperature 350°C, sheath gas 45 arb, auxiliary gas 10 arb, mass spectrum collection range 80–800 m/z, resolution 70,000, S-Lens RF level 55. Potential biomarker ions were detected using MS/MS; the collision energy was optimized automatically according to the ion conditions.

The stability and repeatability of the method from sample pretreatment to detection were monitored by inserting a biological QC sample into the UPLC-Q-Exactive MS/MS spectral analysis detection sequence and investigating its principal component analysis (PCA) results. Before detecting a large number of plasma samples, the QC samples were tested twice consecutively to balance the system; approximately six samples were inserted in each interval for testing.

### Data processing

ProteoWizard software (https://proteowizard.sourceforge.io/) was used to convert the raw file format into the “mzXML” format recognized by the XCMS software (http://metrin.scripps.edu/download/) to filter the peak mass spectrometry data. The positive- and negative-ion modes of all serum samples were imported into R software (v3.5.2) to obtain the retention time, mass charge ratio, and peak strength for multivariate analysis. The three-dimensional data matrix was converted to a “.csv” format file using Microsoft Excel and then imported into MetaboAnalyst 4.0 (http://www.metaboanalyst.ca) software for metabolomics data preprocessing and multivariate analysis. A t-test, analysis of variance, PCA, partial least-squares discriminant analysis (PLS-DA), and orthogonal partial least-squares discriminant analysis (OPLS-DA) were used to complete the data matrix integrity test, missing value estimation, data filtering and standardization, and other preprocessing steps (Li et al., 2022). Through PLS-DA projection of variable importance (VIP) and Volcano Plot of fold change (FC), variables with a VIP >1 and | log2 (FC) | > 1 were were selected as promising biomarkers. Combined with t-test, variables with P > 0.05 were removed to obtain the final difference variables.

Based on the potential metabolic markers’ molecular weights, we searched for candidate results using the Human Metabolome Database (HMDB, http://www.hmdb.ca), Kyoto Encyclopedia of Genes and Genomes (KEGG, http://www.gene.jp/kegg/), and other databases. The mass spectrum structures of the different compounds were analyzed, referring to the relevant literature. The intelligent analysis of metabolic pathways used the relevant database in MetaboAnalyst 4.0 software (Ju et al., 2021).

### Statistical analysis

All statistical analysis was performed using SPSS 25.0, according to the differences in indicators and data, including the chi-squared test, t-test, one-way ANOVA, rank sum test, and partial least squares regression analysis. All data are expressed in $\bar{X}$ ± S mean ± standard deviation. P > 0.05 indicates no statistical difference; P < 0.05 indicates a significant statistical difference; P < 0.01 indicates a very significant statistical difference.

## Results

### Participant characteristics

The study included 90 participants: 16 males and 14 females in group 1 (average age 74.12 ± 4.12 years), 18 males and 12 females in group 2 (average age 73.33 ± 4.25 years) and 17 males and 13 females in control group (average age 72.93 ± 4.15 years). Among the baseline data of the three groups, no statistical difference was found in average age, body mass index, and sex. During the 12-week intervention period, no shedding cases occurred in the intervention group.

### Blood pressure and safety results

**FIGURE 1:**
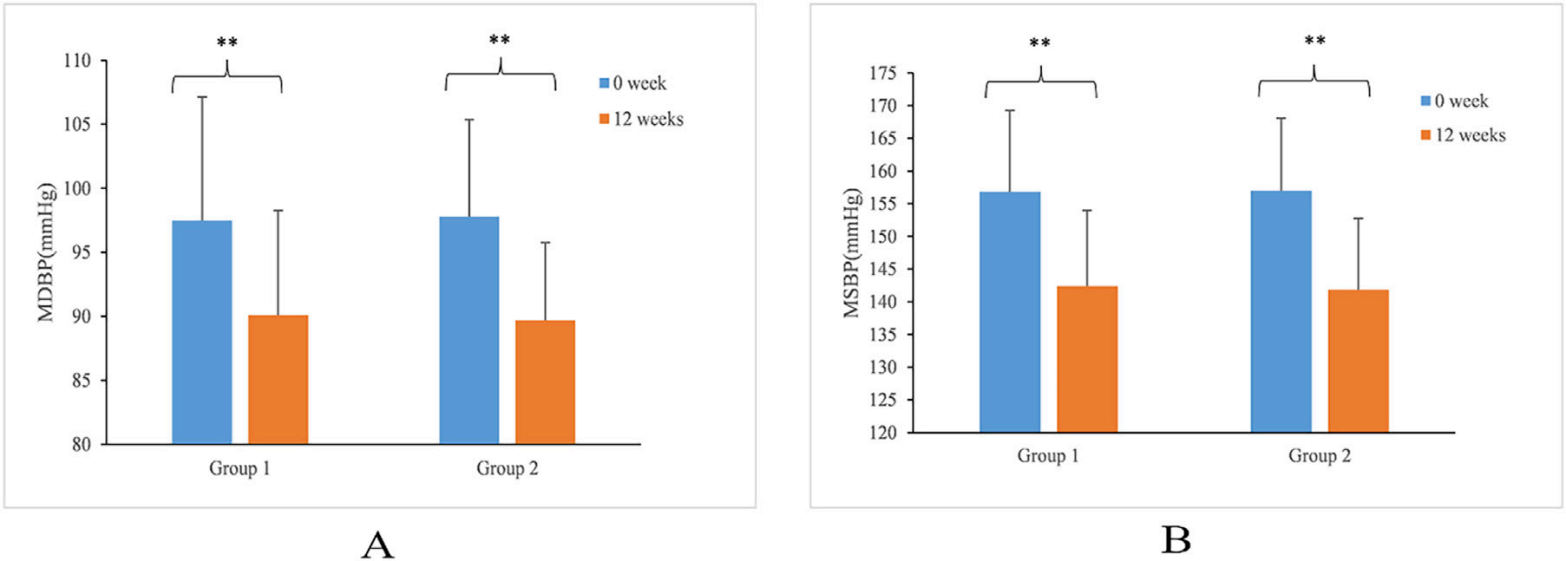
The change trend of MSBP **(A)** and MDBP **(B)** after 12 weeks of administration. **P < 0.01.

Before the intervention, the mean SBP and DBP in group 1 (156.78 ± 12.44 and 97.47 ± 9.66 mmHg, respectively) showed no difference compared with group 2 (156.99 ± 11.08 and 97.78 ± 7.57 mmHg, respectively). Compared with before intervention, group 1 (142.36 ± 11.62 and 90.08 ± 8.19 mmHg, respectively) and group 2 (141.81 ± 10.95 and 89.68 ± 6.07 mmHg, respectively) showed significantly decreased SBP and DBP after 12 weeks of medication (Figure 1). No significant difference was found between group 1 and 2.

Before and after the intervention, groups 1 and 2 showed normal routine blood tests (white blood cells, red blood cells, hemoglobin, and platelet counts) and liver and kidney function indices (alanine aminotransferase, aspartate aminotransferase, blood urea nitrogen, and serum creatinine). After the t-test, no statistical difference was observed between the two groups before and after the intervention.

### Plasma metabolic profile

**FIGURE 2:**
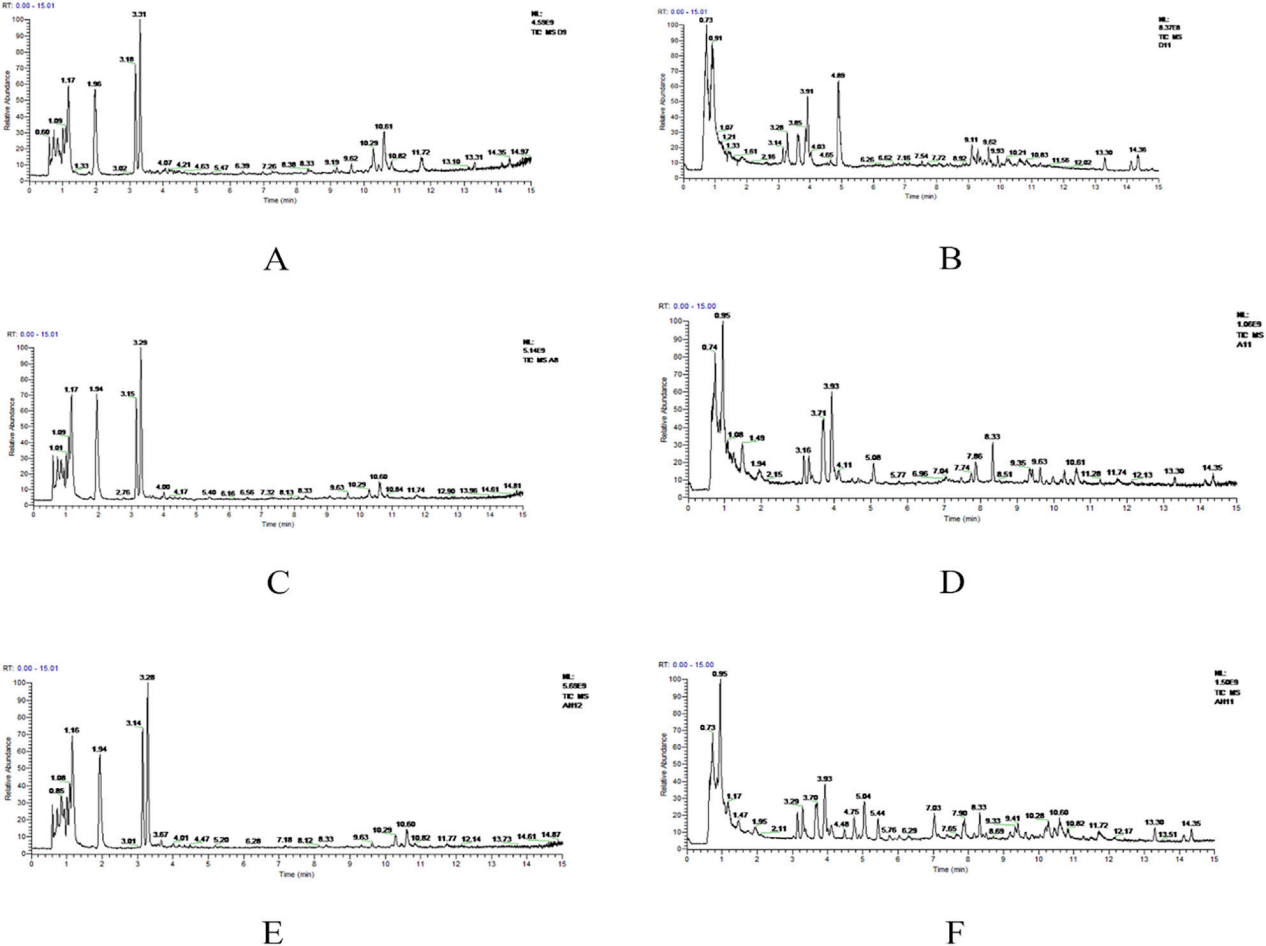
Total ion chromatogram (TIC) of the control, group 1, and YJG group in the positive-ion **(A, C, E)** and negative-ion modes **(B, D, F)**.

**FIGURE 3:**
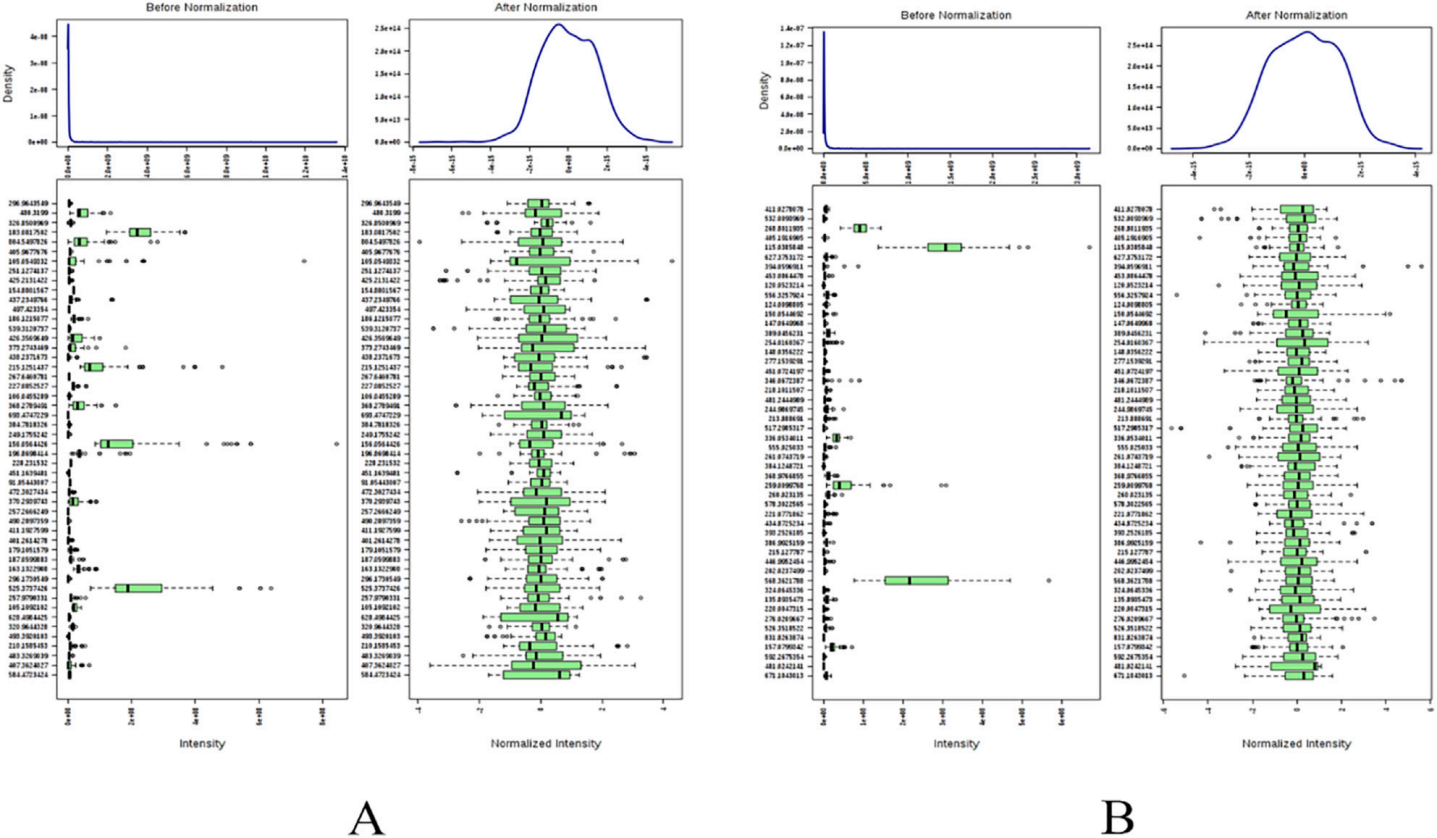
Data standardization in the positive-ion **(A)** and negative-ion modes **(B)**.

The metabolic profiles of the plasma samples under positive and negative ions were tested in healthy individuals and older adults with hypertension before and after YJG intervention. The total ion chromatogram (TIC) in the two modes showed differences among the chromatographic peaks of the three groups (Figure 2). The positive-ion data yielded 1165 variables; 1287 variables were obtained from the negative-ion data. The MetaboAnalyst 4.0 data normalization module was used to standardize and normalize the raw data generated by positive- and negative-ion detection. The original data matrix generated in the two ion-detection modes was skewed; the data had a symmetric normal distribution after data standardization (Figure 3).

### Method validation

**FIGURE 4:**
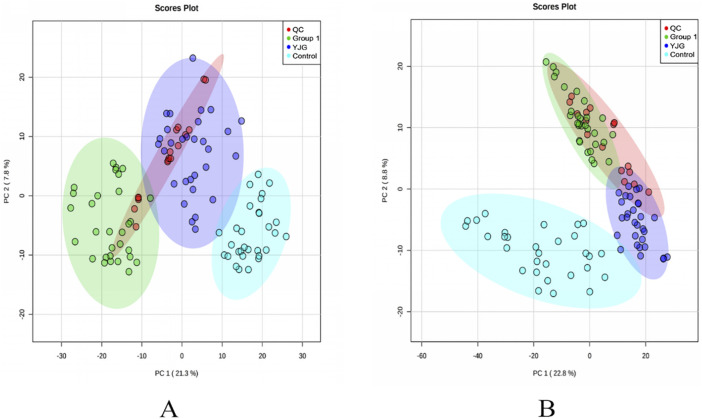
Principal component analysis (PCA) two-dimensional score map of the control, group 1, QC, and YJG group. **(A)**, positive-ion mode; **(B)**, negative-ion mode).

PCA was performed on the data matrix containing the QC samples to determine the data’s reliability (Figure 4). Under the positive- and negative-ion detection modes, QC samples were concentrated within a 95% confidence interval, indicating reliable data quality and good stability and repeatability for the two detection methods.

### Multivariate statistical analysis

**FIGURE 5:**
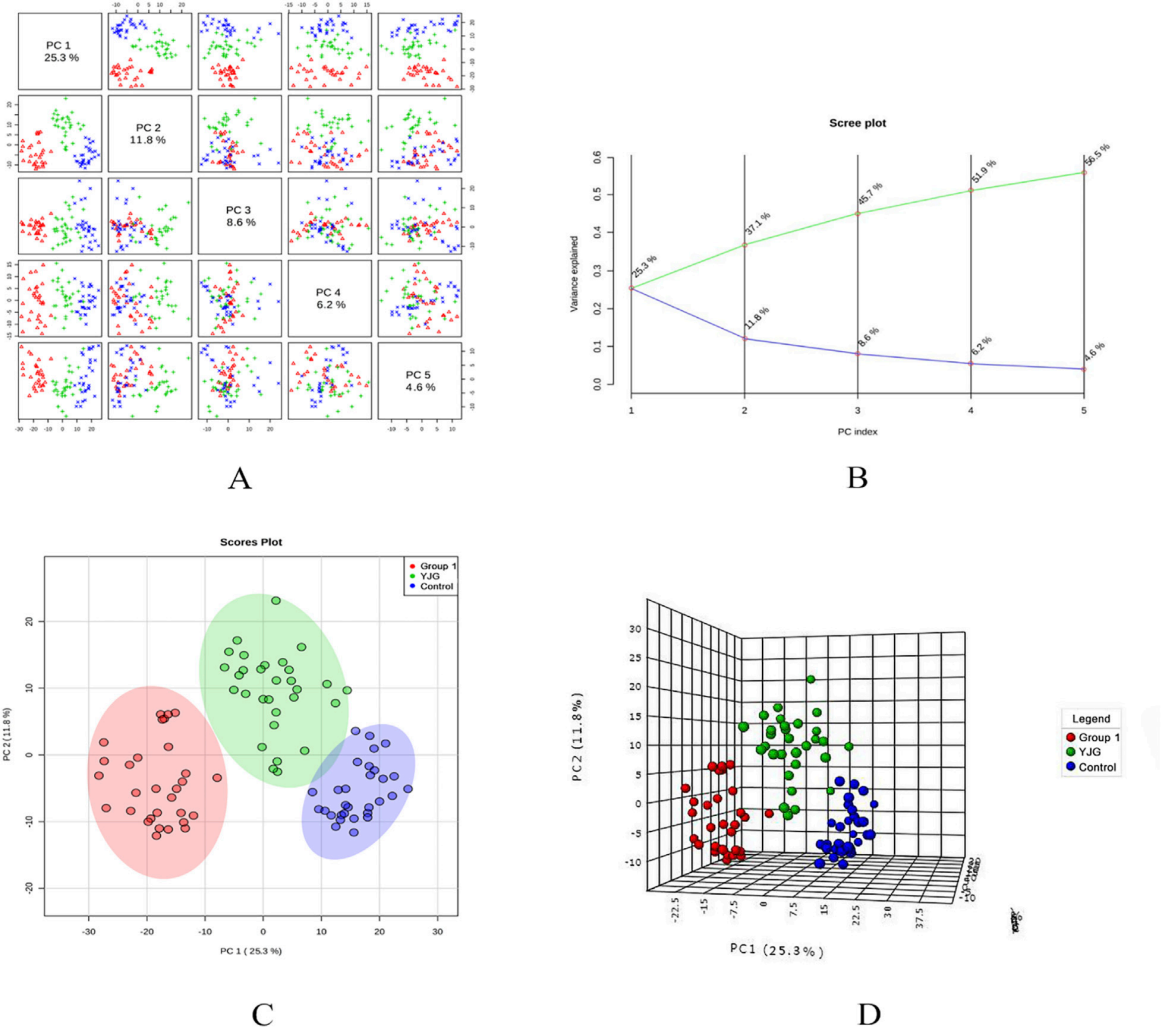
Paired scores **(A)**, variance interpretation of gravel **(B)**, two-dimensional score map **(C)**, and three-dimensional score map **(D)** of PCA in the positive-ion mode of the control, group 1, and YJG group.

**FIGURE 6:**
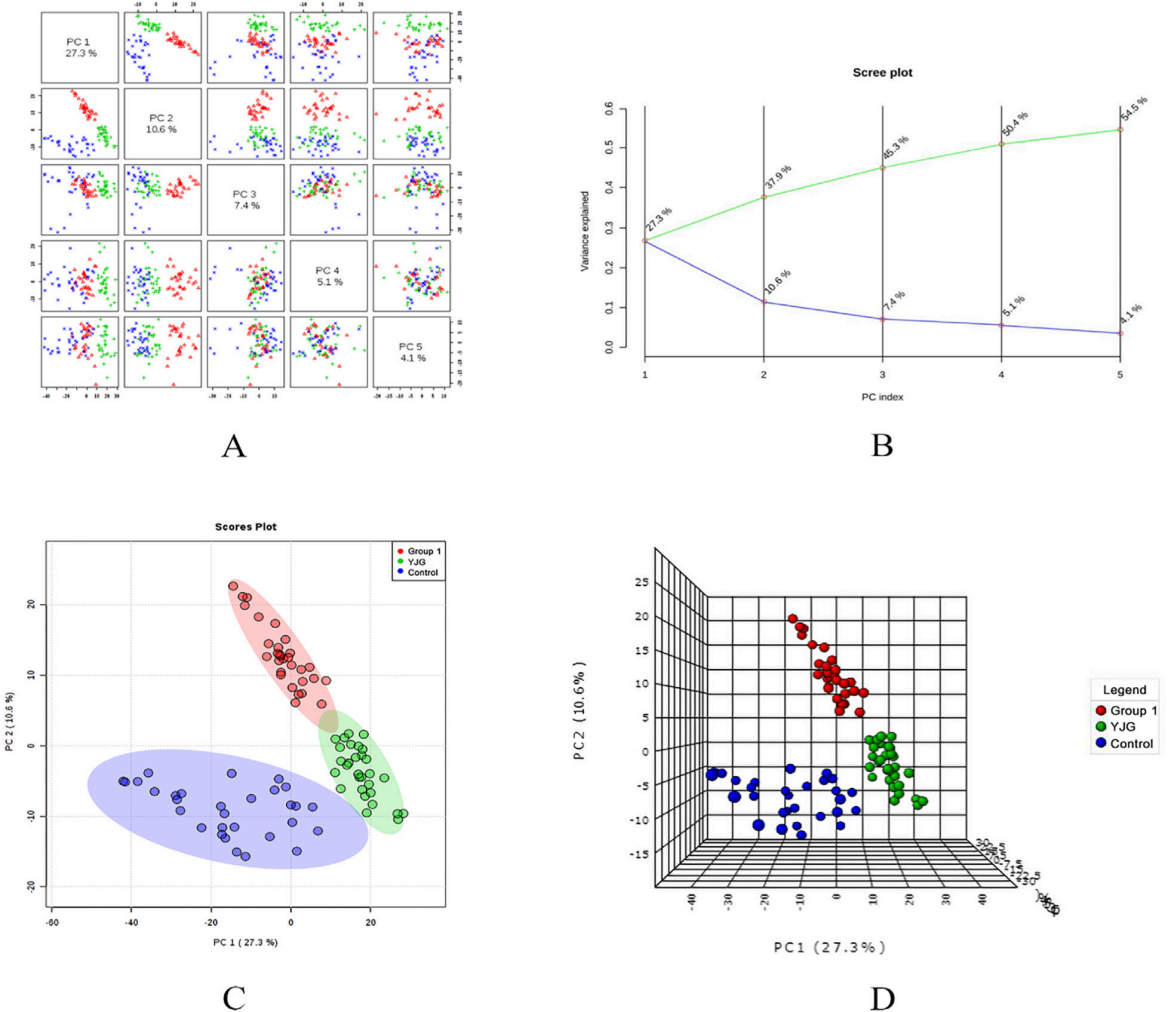
Paired scores **(A)**, variance interpretation of gravel **(B)**, two-dimensional score map **(C)**, and three-dimensional score map **(D)** of PCA in the negative ion mode of the control, group 1, and YJG group.

PCA modeling was performed on the standardized data matrix of the control, group 1, and YJG group (group 1 after YJG intervention); a grouping trend was observed (Figures 5, 6). Five principal components were extracted from the PCA model in the positive-ion mode, with a cumulative interpretation of 56.5%. Five principal components were extracted from the PCA model in the negative-ion mode, with a cumulative interpretation of 54.5%. Two-dimensional and three-dimensional PCA scores showed that the metabolites of the three groups had obvious classification effects. This finding indicated that the serum metabolic status of hypertensive patients was significantly altered compared to healthy participants. The distance between the scatter plots of the samples from group 1 (after intervention) and the control group indicated that older adults with hypertension tended to return to a normal state of health after YJG intervention.

**FIGURE 7:**
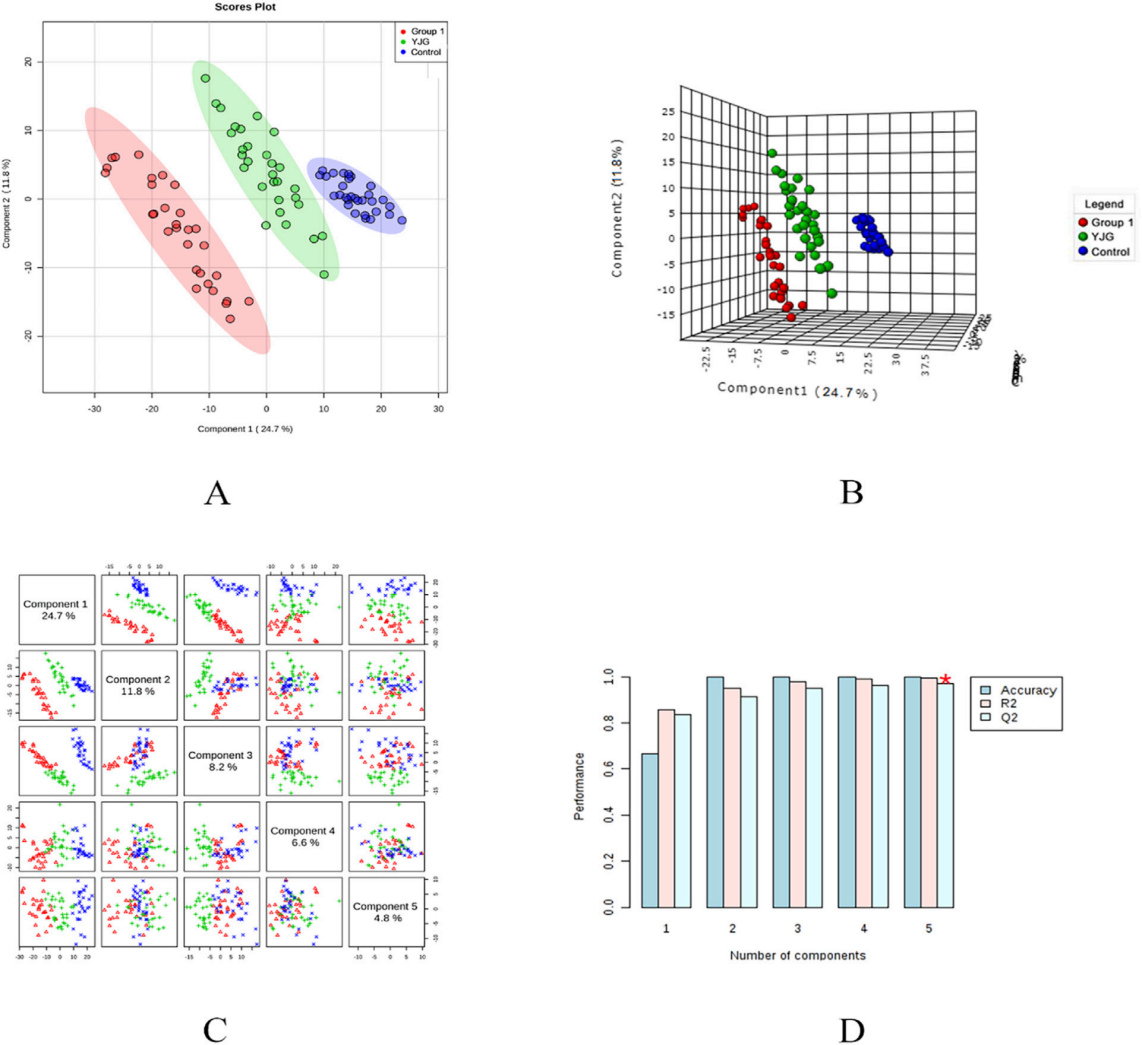
Two-dimensional score map **(A)**, three-dimensional score map **(B)**, paired scores **(C)**, and efficiency evaluation **(D)** of the PLS-DA in the positive ion mode of the control, group 1, and YJG group.

**FIGURE 8:**
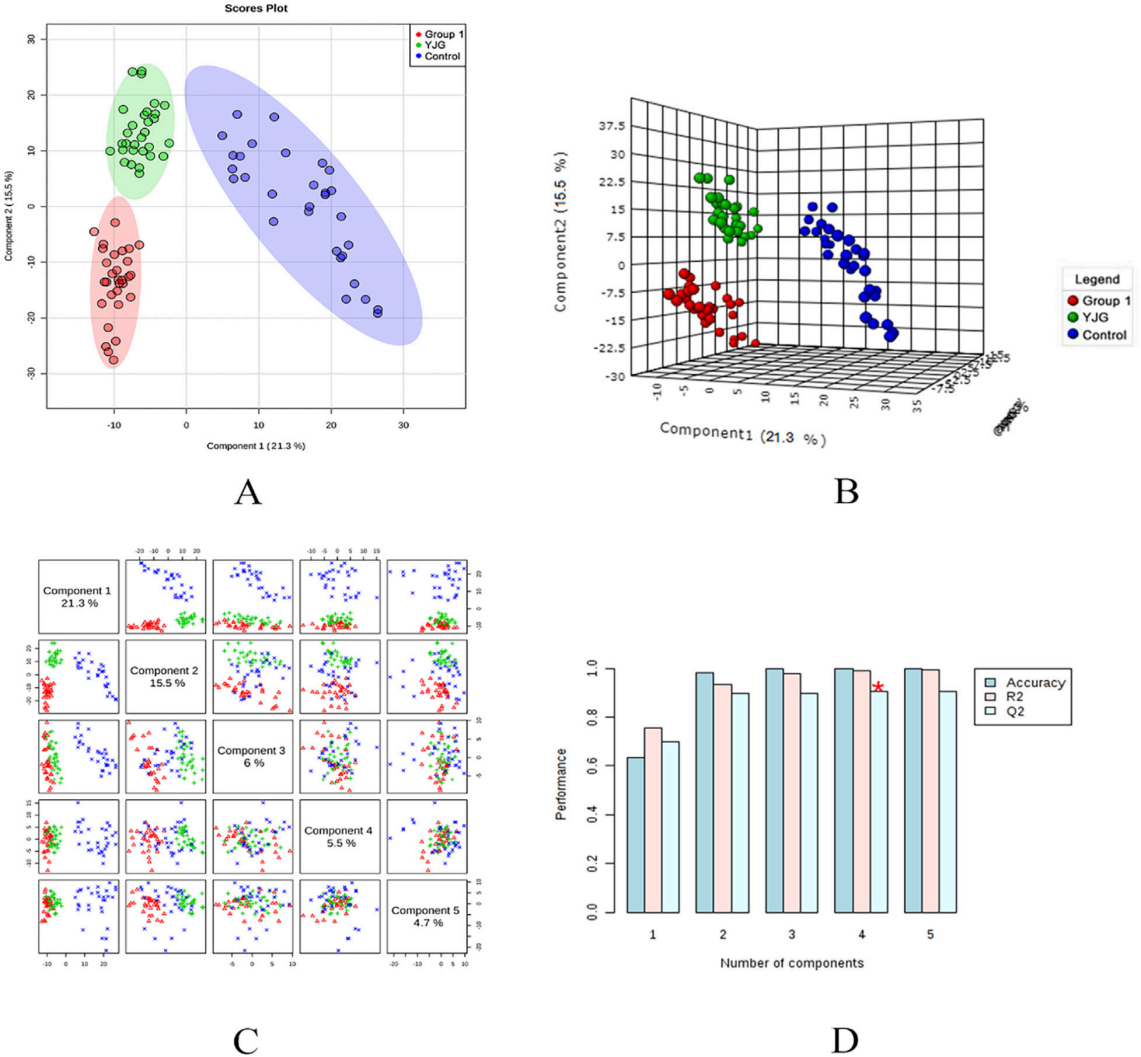
Two-dimensional score map **(A)**, three-dimensional score map **(B)**, paired scores **(C)**, and efficiency evaluation **(D)** of the PLS-DA in the negative ion mode of the control, group 1, and YJG group.

The PLS-DA model was established to predict the difference further; a supervised discriminant analysis method was used to distinguish different groups (Figures 7, 8). The two-dimensional and three-dimensional PLS-DA scores in the positive- and negative-ion detection modes showed the three groups of samples had obvious separation effects. This result showed differences in endogenous metabolic patterns among the three groups of samples. Furthermore, compared with before the intervention, group 1 showed a tendency to approach the control group after the YJG intervention. This result suggested that YJG had a significant ameliorative effect on older adults with hypertension. Q2 estimates a model’s predictive ability and is calculated via 10-fold cross-validation. The PLS-DA model showed stability and reliability in the positive-ion mode (R2 = 99.54%, Q2 = 96.89%, accuracy = 100%) and negative-ion mode (R2 = 99.04%, Q2 = 90.58%, and accuracy = 100%). No overfitting was discovered based on the evaluation results of the permutation test.

### Differential biomarker screening

First, the control group, group 1, and YJG group were analyzed using PLS-DA to select variables contributing to the binding model (VIP>1) to obtain their retention time and quality-to-kernel ratio information. One-way ANOVA was performed on the selected variables to remove those with P > 0.05. Preliminary screening for differential variables as potential metabolic markers identified 363 in the positive-ion and 399 in the negative-ion modes.

**FIGURE 9:**
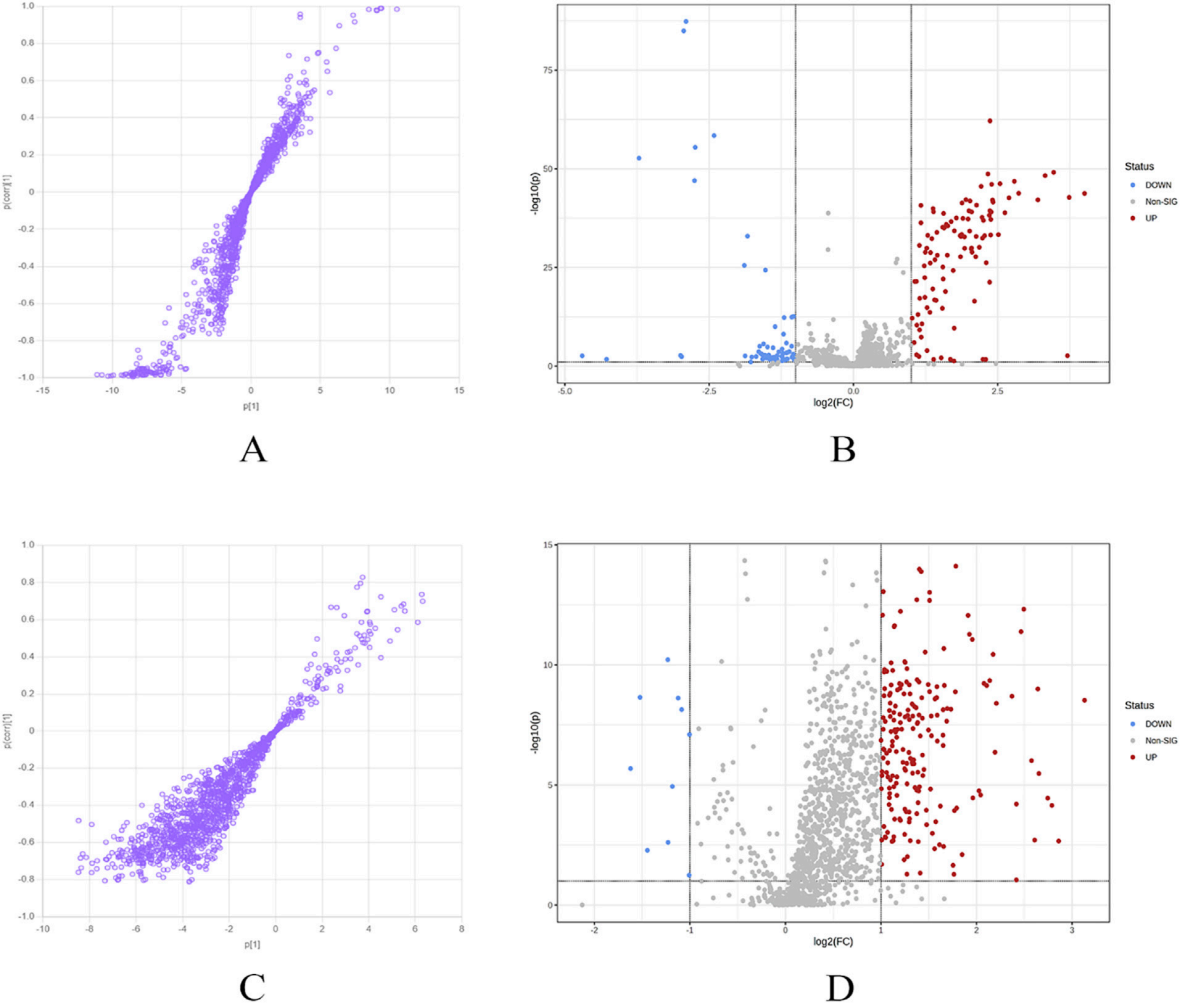
S-plot **(A)**, volcano plot **(B)** in the positive mode, S-plot **(C)**, volcano plot **(D)** in the negative mode of group 1 and YJG group.

Further analysis was conducted on the metabolites of group 1 before and after YJG intervention. Using the S-Plot generated based on OPLS-DA to screen for the most promising biomarkers (Figure 9), 257 variables with |p | >5 and |p (corr) >0.2 were selected in the positive-ion mode; 579 variables with |p | >5 and |p (corr) | >0.4 were selected in the negative-ion mode. A volcano plot was used for the difference analysis. In both the positive- and negative-ion modes, variables with | log2 (FC) | >1 and P < 0.05 in the data matrix were selected as the most promising biomarkers (Figure 9). We selected the variables in the PLS-DA that contributed to the binding model (VIP >1). A combination of fold change and t-tests was used to screen for significant differential variables as differential metabolic markers for the remaining differential variables. The positive-ion mode yielded 69 variables and the negative-ion mode yielded 48 variables.

Duplicate results from the two screenings were selected. The differential metabolic markers were imported into HMDB, METLIN, KEGG, and other public biological databases for comparison with relevant literature. The compounds were analyzed and confirmed using secondary mass spectrometry. Ultimately, 30 differential metabolic markers were identified (23 and 7 in the positive- and negative-ion modes, respectively) (Table 1).

**TABLE 1:** Metabolic marker information.

No.	m/z	Identification results	Plus-minus	Chemical formula	KEGG	HMDB	Trend
1	315.2529	12,13-Dhome	+	C18H34O4	C14829	HMDB0004705	↑
2	911.5518	PI(20:3(8Z,11z,14Z)/20:3(5Z,8Z,11z)	+	C49H83O13P	C00626	HMDB0009891	↓
3	335.2543	Tetrahydrodeoxycorticosterone	+	C21H34O3	C13713	HMDB0000879	↑
4	371.2428	Thromboxane B2	+	C20H34O6	C05953	HMDB0003252	↓
5	752.5561	PE(P-18:1(1Z)/20:4(5Z,8Z,11Z,14Z))	+	C43H78NO7P	C00350	HMDB0005779	↓
6	733.6184	SM(d18:0/18:0)	+	C41H85N2O6P	C00550	HMDB0012087	↑
7	309.2794	Eicosadienoic acid	+	C20H36O2	C16525	HMDB0005060	↑
8	331.2537	Docosapentaenoic acid (22n-3)	+	C22H34O2	C16513	HMDB0006528	↓
9	816.5844	PC(22:6(4Z,7Z,10Z,13Z,16Z,19Z)/P-18:1(9Z)	+	C48H82NO7P	C00157	HMDB0008754	↓
10	818.5925	PC(22:6(4Z,7Z,10Z,13Z,16Z,19Z)/P-18:0)	+	C48H84NO7P	C00157	HMDB0008752	↓
11	457.3416	Stigmasterol	+	C58H108N2O18	C05442	HMDB0000937	↑
12	481.4256	5-b-Cholestane-3a,7a,12a-triol	+	C27H48O3	C05454	HMDB0001457	↓
13	496.4254	TG(18:2(9Z,12Z)/18:2(9Z,12Z)/20:1(11Z))	+	C59H104O6	C00422	HMDB0005473	↓
14	501.373	Hyperforin	+	C35H52O4	C07608	HMDB0030463	↑
15	524.4567	TG(20:0/20:1(11Z)/20:4(5Z,8Z,11Z,14Z))	+	C63H112O6	C00422	HMDB0005419	↓
16	561.3871	Ganglioside GA2 (d18:1/20:0)	+	C18H34O4	C06135	HMDB0004892	↑
17	577.3719	LysoPC(16:1(9Z)/0:0)	+	C24H48NO7P	C04230	HMDB0010383	↓
18	584.4722	Gamma-linolenic acid	+	C18H30O2	C06426	HMDB0003073	↑
19	673.5401	DG(18:2(9Z,12Z)/20:4(5Z,8Z,11Z,14Z)/0:0)	+	C41H68O	C00165	HMDB0007257	↓
20	716.5594	PC(16:0/16:0)	+	C29H48O	C00157	HMDB0000564	↓
21	717.5541	PE(16:1(9Z)/P-18:1(9Z))	+	C39H74NO7P	C00350	HMDB0008985	↓
22	777.5880	SM(d18:0/20:2(11Z,14Z))	+	C43H83N2O6P	C00550	HMDB0013465	↑
23	820.5884	PC(16:1(9Z)/P-18:1(11z)	+	C42H80NO7P	C00157	HMDB0008029	↓
24	279.2329	Linoleic acid	−	C18H32O2	C01595	HMDB0000673	↑
25	281.2486	Elaidic acid	−	C14H26C18H34O2	C01712	HMDB0000573	↑
26	303.2329	Arachidonic acid	−	C20H32O2	C00219	HMDB0001043	↓
27	327.2305	8,11,14-Eicosatrienoic acid	−	C20H34O2	C03242	HMDB0002925	↑
28	327.2540	Vaccenic acid	−	C18H34O2	C08367	HMDB0003231	↑
29	445.3323	Calcidiol	−	C27H44O2	C01561	HMDB0003550	↑
30	562.3150	LysoPC(18:3(6Z,9Z,12Z))	−	C26H48NO7P	C04230	HMDB0010387	↓

“+” indicates the positive-ion mode, “−”indicates the negative-ion mode. Codes indicate the compound’s identification number in the KEGG, or HMDB, database. Arrows indicate the trend in biomarker levels in the treatment vs model group as rising “↑” or decreasing “↓”.

### Metabolic pathway analysis

**FIGURE 10:**
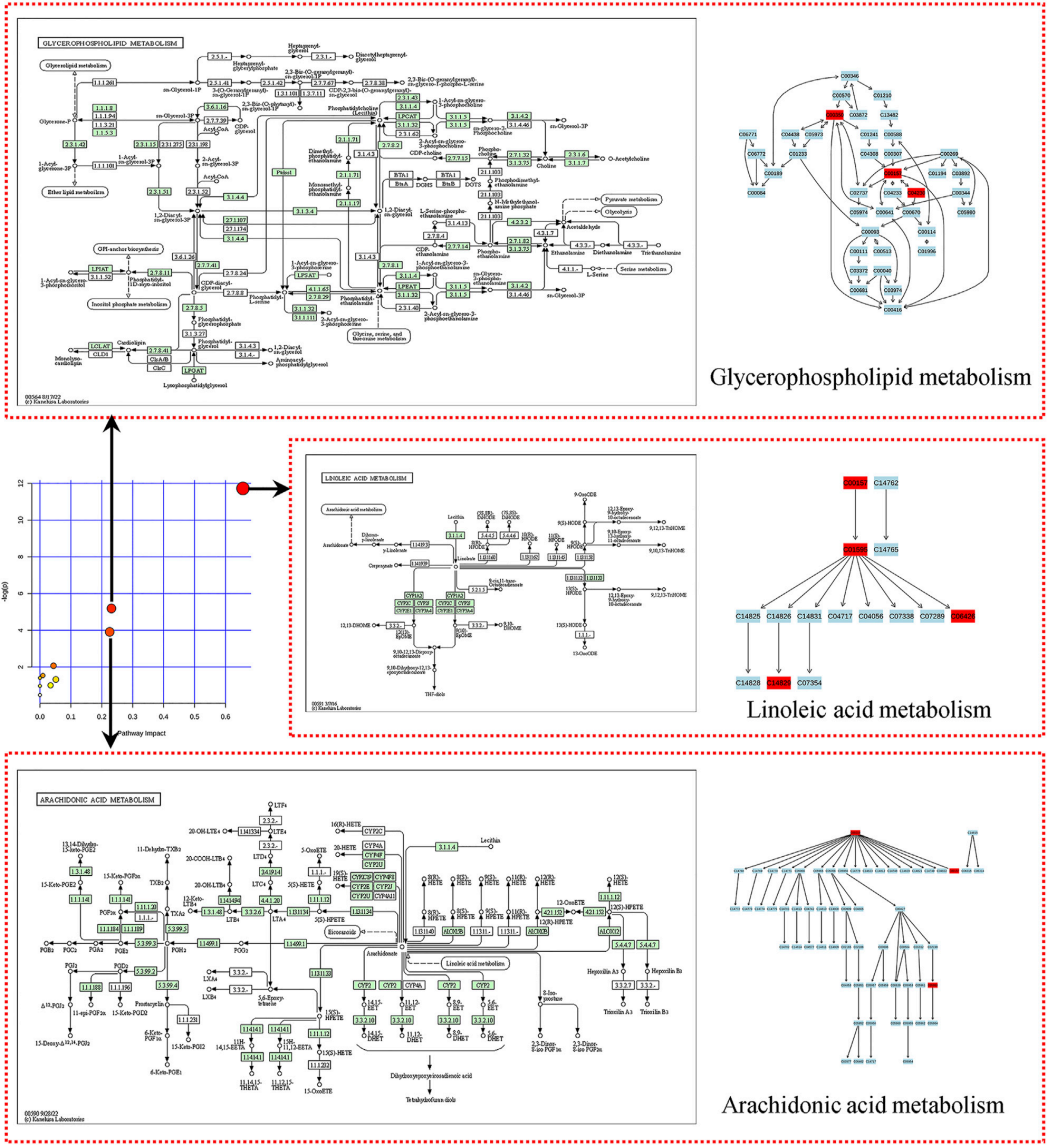
Summary of metabolic pathways (Arrows pointing to differential metabolic pathways, including linoleic acid, arachidonic acid, and glycerophospholipid metabolism).

To investigate the relationship between metabolites and YJG, the HMDB ID numbers of the identified differential biomarkers were entered into the pathway analysis module provided by MetaboAnalyst 4.0. A metabolic pathway summary chart (Figure 10) was drawn to generate a metabolic pathway analysis summary table (Table 2). The pathways with influence values greater than 0.1 in the pathway topology analysis were used as potential differential metabolic pathways; three metabolic pathways were identified, including arachidonic acid metabolism, linoleic acid metabolism, and glycerophospholipid metabolism.

**TABLE 2:** Metabolic pathway analysis.

No	Metabolic pathway	Total	Hits	Expected	Impact
1	Arachidonic acid	62	3	0.56668	0.2255
2	Linoleic acid	15	4	0.1371	0.65625
3	Glycerophospholipid	39	3	0.35646	0.23123

Expected: the expected value of the pathway obtained by topological analysis. Hits: number of exact matches in the uploaded marker data. Impact: influence value of the pathway obtained via topological analysis.

## Discussion

Although more drugs are being developed to treat hypertension, the control rate of blood pressure and improvement of clinical symptoms remains unsatisfactory. Many recent studies have identified and compared metabolites in the blood, urine, or tissues to identify their pathogenesis, potential biomarkers, effects of drug interventions, and mechanisms of action in human or animal models. Our preliminary research first screened 20 characteristic biomarkers and 6 related metabolic pathways in older spontaneously hypertensive rats (SHR) compared to Wistar Kyoto rats. The linoleic acid levels of SHR were significantly reduced; the levels of phosphatidyl ethanolamine (PE) and lysophosphatidylcholine (LPC) were significantly increased (Zhang et al., 2022). In addition, YJG was used to treat older SHR, significantly decreasing blood pressure. Twenty-six metabolic targets and four targeted metabolic pathways were analyzed, including linoleic acid metabolism, glycerophospholipid metabolism, arginine and proline metabolism, and steroid hormone biosynthesis (Zhang et al., 2021). This clinical trial explored the effects of YJG on blood pressure and metabolites in older adults with hypertension. After taking YJG, blood pressure decreased significantly, and the metabolic status of some compounds tended to return to normal. We found changes in 30 plasma metabolites related mainly to the metabolic pathways for linoleic acid, arachidonic acid, and glycerophospholipids.

Linoleic acid is an essential polyunsaturated fatty acid for humans and can reduce the risk of major cardiovascular events (Marklund et al., 2019). Studies have demonstrated a correlation between linoleic acid and hypertension. A study in Japanese men on the association between plasma phospholipid fatty acids and hypertension found that the plasma levels of linoleic acid were significantly lower in individuals with hypertension than in healthy controls; an increase in the mean linoleic acid level (from 15 to 21 mol%) decreased the incidence of hypertension to 39% (Tsukamoto and Sugawara, 2018). An L-shaped nonlinear association exists between total polyunsaturated fatty-acid intake and the incidence of hypertension among American adults; a linoleic acid intake >18 mg/kg/day significantly reduced the incidence of hypertension (Ni et al., 2022). YJG treatment increased plasma linoleic acid levels in our study. Linoleic acid can improve the bioavailability of NO and reduce the participation of COX-2-derived vasoconstrictor prostaglandins; this pathway may mediate how YJG exerts its antihypertensive effects (Nunes et al., 2018). Gamma-linolenic acid (GLA) is metabolized and converted from linoleic acid in the body; its production decreases with age. GLA is enriched in borax oil and can reduce blood pressure in postmenopausal hypertensive women (Da et al., 2021). This moderate effect may be due to interference of prostaglandins and thromboxanes resulting from the metabolism of borage oil, leading to vasodilation and renovascular regulatory improvement. GLA also increases the activity of P450 epoxygenase and enhances the synthesis of vasodilatory epoxyeicosatrienoic acids, lowering blood pressure (Yu et al., 2006).

GLA is initially catalyzed by Delta-6 desaturase to convert it into dihomo-γ-linolenic acid, and then further desaturated by Delta-5 desaturase to form arachidonic acid. As a downstream product, arachidonic acid content should be increased when linolenic acid and GLA content are upregulated; however, it was significantly downregulated after YJG administration, indicating that YJG plays a strong role in reducing arachidonic acid. Arachidonic acid is released by phospholipids in the cell membrane and metabolized through the cyclooxygenase, cytochrome P450, and lipoxygenase pathways, playing an important role in regulating vascular tension and cardiovascular complications (Zhou et al., 2021b). Higher arachidonic acid levels are associated with a greater incidence of hypertension. The 1.5 times elevation in the mean arachidonic acid level (from 5.68 to 8.47 mol%) causes a two-fold increase in the incidence of hypertension (Tsukamoto and Sugawara, 2018). Arachidonic acid and its metabolites are crucial in endothelium-dependent contraction (Edwards et al., 2020). The reduction in SBP and DBP by hydrochlorothiazide was significantly associated with arachidonic acid, an important metabolomics feature in the netrin signaling pathway (Shahin et al., 2016). Arachidonic acid is converted into prostaglandin H_2_ by cyclooxygenase, generating thromboxane A_2_ through thromboxane A synthase, ultimately hydrolyzing it into thromboxane B_2_. He identified thromboxane B_2_ in a non-targeted metabonomic study of human hypertension (He et al., 2020). Thromboxane B_2_ is an inactive metabolite of thromboxane A_2_, which decreases after treatment with YJG, corresponding to decreased thromboxane A_2_ levels in the body. Thromboxane A_2_ induces platelet aggregation and vasoconstriction; an increase in its concentration may damage the contraction function of the aortic ring wall, inducing an increase in blood pressure (Hu et al., 2021). Thromboxane A_2_ biosynthesis was enhanced in patients with hypertensive and SHR mice. Aspirin administration caused a selective inhibition of systemic TXA_2_ biosynthesis and leads to a reduction in blood pressure (D'Agostino et al., 2021; Li et al., 2022). Prostaglandin E2 and Prostaglandin I2 have been reported to have vasodilatory effects and can lower blood pressure. However, this study did not observe significant changes in other prostaglandin levels after Yishenjiangya (YSJ) intervention, thus the impact on other prostaglandins remains unclear.

Glycerophospholipid metabolism changes in the early stages of hypertension; LPC and PE are predictive metabolites of hypertension (Liu et al., 2022). Different association magnitudes have been observed between lipidomic subclasses and hypertension risk. The PE subclass is the most significant among nine hypertension-associated lipidomic subclasses in a population of middle-aged and older adult Chinese individuals (Niu et al., 2022). LPC is a major component of oxidative damage to low-density lipoprotein and can induce inflammation and vascular dysfunction in cardiovascular diseases (Liu et al., 2020). One study found a significant increase in fatty acids, phosphatidylcholine (PC), and LPC levels in patients with hypertension (Ke et al., 2018). LPC is an important trigger of endothelial dysfunction and attenuates acetylcholine-induced relaxation by inducing proconstricting prostanoids and superoxide anions (Rao et al., 2013). LPC also induces neuronal NO synthase uncoupling and its phosphorylation, a consequent reduction in NO and H_2_O_2_ production, and increased superoxide production due to ERK1/2 upregulation in human and murine endothelial cells, thereby increasing phenylephrine-induced vasoconstriction (Campos-Mota et al., 2017). LPC is independently associated with decreased SBP; its content decreases after chlorothiazide administration, consistent with the results of using YJG (Huang et al., 2022).

Among the differential metabolites screened, PC was the only metabolite that is involved in three metabolic pathways and also regulates blood pressure. PC contains various fatty acids and is a source of linoleic acid. Arachidonic acid and LPC are downstream metabolites of PC and are produced by phospholipase A2. PC content decreased after YJG intervention in our study, possibly explaining the downregulation of related metabolites. PC is the most abundant phospholipid in mammalian cells. PC is a component of cell and organelle membranes, participates in the assembly and secretion of lipoproteins, functions as a precursor of signaling molecules and inflammatory mediators, and is important in the occurrence and development of cardiovascular diseases such as hypertension and coronary heart disease (Jiang et al., 2022).

The novelty of this study lied in focusing on older adults, a high-risk group for hypertension, observing the clinical efficacy of YJG and exploring the target and pathway of YJG’s action on hypertension through metabolomics methods. The results help explain YJG’s antihypertensive effect, provide a basis for its clinical application in older adults with hypertension, and provide new directions for treating hypertension. However, our study had some limitations. First, only plasma was collected for differential metabolite analysis; urine, feces, and other samples could not be collected simultaneously for analysis to identify more accurate changes in small metabolites. Second, we conducted non-targeted metabolomics studies to comprehensively and systematically reflect metabolic characteristics but failed to provide more accurate targeted omics validation of the results, possibly leading to instability in the results. In addition, metabolite detection technology has many requirements and complex procedures and is susceptible to multiple factors. Single metabolomics research has limitations in explaining the occurrence, development, and outcomes of hypertension. Transcriptomics and proteomics will be combined to resolve the detailed mechanism and regulatory network of YJG’s effect on hypertension.

## Conclusion

Our study demonstrated that YJGs are safe and can reduce blood pressure in older adults with hypertension. UPLC-Q-TOF-MS-based plasma metabolomics profiling and multivariate statistical analyses evaluated the trends of metabolic networks in older adults with hypertension. Thirty potential biomarkers were identified, associated mainly with the metabolism of linoleic acid, arachidonic acid, and glycerophospholipid. This study provides a scientific basis for using TCM to treat hypertension in older adults. Our findings provide novel insights into YJG’s efficacy and how it reduces blood pressure and an experimental basis for the clinical application of YJG in older adults with hypertension.

## Data Availability

The original contributions presented in the study are included in the article/supplementary material, further inquiries can be directed to the corresponding author.
